# Association between Impella device support and elevated rates of gout flares: a retrospective propensity-matched study

**DOI:** 10.1186/s41927-024-00380-z

**Published:** 2024-02-29

**Authors:** Jorge Sinclair De Frías, Shahin Isha, Lorenzo Olivero, Lekhya Raavi, Sai Abhishek Narra, Smit Paghdar, Sadhana Jonna, Parthkumar Satashia, Rachel Hannon, Jessica Blasavage, Layton White, Titilope Olanipekun, Pankaj Bansal, Sean Kiley, Juan Carlos Leoni, Jose Nativí, Melissa Lyle, Mathew Thomas, Basar Sareyyupoglu, Si Pham, Michael Smith, Pablo Moreno Franco, Parag Patel, Devang Sanghavi

**Affiliations:** 1 Department of Critical Care Medicine, Mayo Clinic, 4500 San Pablo Road S, 32224 Jacksonville, FL USA; 2 Associate Clinical Consultant, Abiomed, Jacksonville, FL USA; 3 Department of Pulmonary and Critical Care Medicine, Brigham and Women’s Hospital, Harvard Medical School, Boston, MA USA; 4 Department of Rheumatology, Mayo Clinic Health System, Eau Claire, WI USA; 5 Division of Advanced Heart Failure and Transplantation, Department of Transplantation, Mayo Clinic, Jacksonville, FL USA; 6 Department of Cardiovascular and Thoracic Surgery, Mayo Clinic, Jacksonville, FL USA; 7 Department of Anesthesiology and Perioperative Medicine, Mayo Clinic, Jacksonville, FL USA

**Keywords:** Gout, Circulatory failure, Cardiogenic shock, Hemolysis, Hemorrhage

## Abstract

**Background:**

Impella is an advanced ventricular assist device frequently used as a bridge to heart transplantation. The association of Impella with increased rates of gout flares has not been studied. Our primary aim is to determine the rates of gout flares in patients on Impella support.

**Methodology:**

A retrospective study was conducted between January 2017 and September 2022 involving all patients who underwent heart transplantation. The cohort was divided into two groups based on Impella support for statistical analysis. In patients receiving Impella support, outcome measures were compared based on the development of gout flares. 1:1 nearest neighbor propensity match, as well as inverse propensity of treatment weighted analyses, were performed to explore the causal relationship between impella use and gout flare in our study population.

**Results:**

Our analysis included 213 patients, among which 42 (19.71%) patients were supported by Impella. Impella and non-Impella groups had similar age, race, and BMI, but more males were in the Impella group. Gout and chronic kidney disease were more prevalent in Impella-supported patients, while coronary artery disease was less common. The prevalence of gout flare was significantly higher in Impella patients (30.9% vs. 5.3%). 42 Impella-supported patients were matched with 42 patients from the non-impella group upon performing a 1:1 propensity matching. Impella-supported patients were noted to have a significantly higher risk of gout flare (30.9% vs. 7.1%, SMD = 0.636), despite no significant difference in pre-existing gout history and use of anti-gout medications. Impella use was associated with a significantly increased risk of gout flare in unadjusted (OR 8.07), propensity-matched (OR 5.83), and the inverse propensity of treatment-weighted analysis (OR 4.21).

**Conclusion:**

Our study is the first to identify the potential association between Impella support and increased rates of gout flares in hospitalized patients. Future studies are required to confirm this association and further elucidate the biological pathways. It is imperative to consider introducing appropriate measures to prevent and promptly manage gout flares in Impella-supported patients.

**Supplementary Information:**

The online version contains supplementary material available at 10.1186/s41927-024-00380-z.

## Introduction

The Impella device represents a significant breakthrough in mechanical circulatory support and has revolutionized the landscape of cardiac care. This catheter-based device is utilized across diverse cardiac care settings, such as high-risk percutaneous coronary intervention, cardiogenic shock after myocardial infarction, cardiac surgery, and cardiomyopathies like severe myocarditis [1]. The Impella device is often placed in the left ventricle, although placement on the right side is also feasible when dealing with right ventricular pump failure. It improves cardiac output, achieving between 2.5 and 5.5 l/min, and optimizes systemic perfusion and coronary blood flow, reducing myocardial oxygen demand and ventricular workload [2].

Among the various Impella devices, two commonly employed options are Impella CP and Impella 5.5, and the selection between them involves nuanced considerations [2]. Impella CP stands out for its rapid placement in the catheterization laboratory but provides limited hemodynamic support with a maximum flow rate of 3.7 l/min. Additionally, the 14 Fr catheter diameter of Impella CP may lead to higher hemolysis rates. Moreover, although accessible through both femoral and axillary arteries, it is predominantly accessed through the femoral artery, limiting patient mobility and participation in pre-transplant rehabilitation. Conversely, Impella 5.5 requires surgical implantation but presents several advantages. It provides a higher level of hemodynamic support with a maximum flow rate of 5.5 l/min. The larger 23 Fr catheter diameter reduces the incidence of hemolysis [3, 4]. Notably, Impella 5.5 is the preferred choice when accessing the axillary artery, enabling patients to engage more actively in pre-transplant rehabilitation. The choice between these devices is therefore intricately tied to their distinct features and the specific needs of the patient.

The Impella device has demonstrated substantial efficacy in improving survival rates among patients experiencing cardiogenic shock prior to undergoing percutaneous coronary intervention due to acute myocardial infarction [2]. Furthermore, the utilization of Impella as a bridging strategy for heart transplant recipients has demonstrated favorable outcomes, characterized by high survival rates and minimal morbidity in the post-transplant period [5]. However, the use of this device poses certain risks, including bleeding, vascular injury, reduced blood flow to the lower limb, stroke, as well as myocardial infarction and hemolysis [2, 6].

Gout is an inflammatory crystal arthropathy commonly resulting from the precipitation of monosodium urate crystals in synovial fluid and tissues that often present as acute flares. These flares are characterized by severe pain, redness, warmth, swelling, and disability and are associated with an increased risk of cardiovascular disease. Common risk factors for acute flares in the hospital setting include diuretic adjustments, surgery, low-dose aspirin, kidney disease, and baseline suboptimal gout treatment [7]. Patients undergoing heart transplantation often present with multiple of these risk factors during their hospital stay. However, an emerging suspicion suggests that Impella devices may serve as an independent risk factor for the onset of gout flares. This suspicion is rooted in our clinical experience, where a notable proportion of Impella-supported patients has been observed to present with gout flares before heart transplantation, a phenomenon less frequently observed in non-Impella-supported patients undergoing heart transplantation. There is a complete absence of published studies exploring this potential association, representing a significant knowledge gap that calls for further research.

This study aims to explore and compare the occurrence of gout flares in heart transplant patients based on the use of Impella support. The insights derived from this study could pave the way for improved patient care protocols, minimizing the risk of complications associated with Impella device management.

## Methods

### Ethics

Our study was deemed exempt by the Mayo Clinic Institution Review Board, under the IRB number 22-008125, and the study title is “Risk of Gout Flares in Patients Managed with an Impella Device.” The need for informed consent was waived by the institutional review board, and the study cohort included patients with prior research authorization. Data anonymity and confidentiality were maintained per standard protocol, and the procedures followed the ethical standards of the committee responsible for human experimentation and the Helsinki Declaration of 1975, as well as the ISHLT statement on transplant ethics.

### Study population and data

Our study included all ≥ 18-year-old hospitalized patients who underwent a heart transplantation at Mayo Clinic, Florida, between January 2017 and September 2022. Patients were divided into two groups based on whether they received Impella support (Impella 5.5 or CP) as a bridge to heart transplantation or not. Clinical variables were obtained from electronic medical records. These variables included demographic characteristics, comorbidities, and outpatient and inpatient medications. Outpatient medications were considered for inclusion if the patient had been taking them up until the day of admission, whereas inpatient medications were eligible for inclusion if they had been administered for a duration of three or more days during the hospitalization. Our primary outcome was rates of gout flares during the admission. Patient were classified as having gout flares when the clinical notes documented occurrences of such flares during their admission. For Impella-supported patients, only gout flare episodes occurring after the insertion of the device were included. In contrast, for the non-Impella group, gout flare episodes were included regardless of when they occurred during the hospitalization period. Secondary outcomes included in-hospital mortality, hospital length of stay (LOS), intensive care unit (ICU) LOS, invasive mechanical ventilation (IMV). Calculations for hospital and ICU length of stay were conducted from admission to discharge, including the pre-operative and post-operative periods. Pre-existing comorbidities (such as chronic kidney disease, coronary artery disease, diabetes mellitus etc.) were identified based on the coded ‘problem list’ available on electronic medical record. Any stage of hypertension or chronic kidney disease, if documented in the problem list, was included and patients were not sub stratified based on the stages.

### Statistical analysis

Statistical analyses were performed on BlueSky v10.3.1 and R Studio (R 4.2.1). Categorical variables were expressed as percentages (%) and compared using the Chi-square test. Continuous variables were expressed as median (1st quartile, 3rd quartile) and compared by Wilcoxon rank sum test for independent samples. A p-value of < 0.05 was considered the cutoff for statistical significance.

**Fig. 1:**
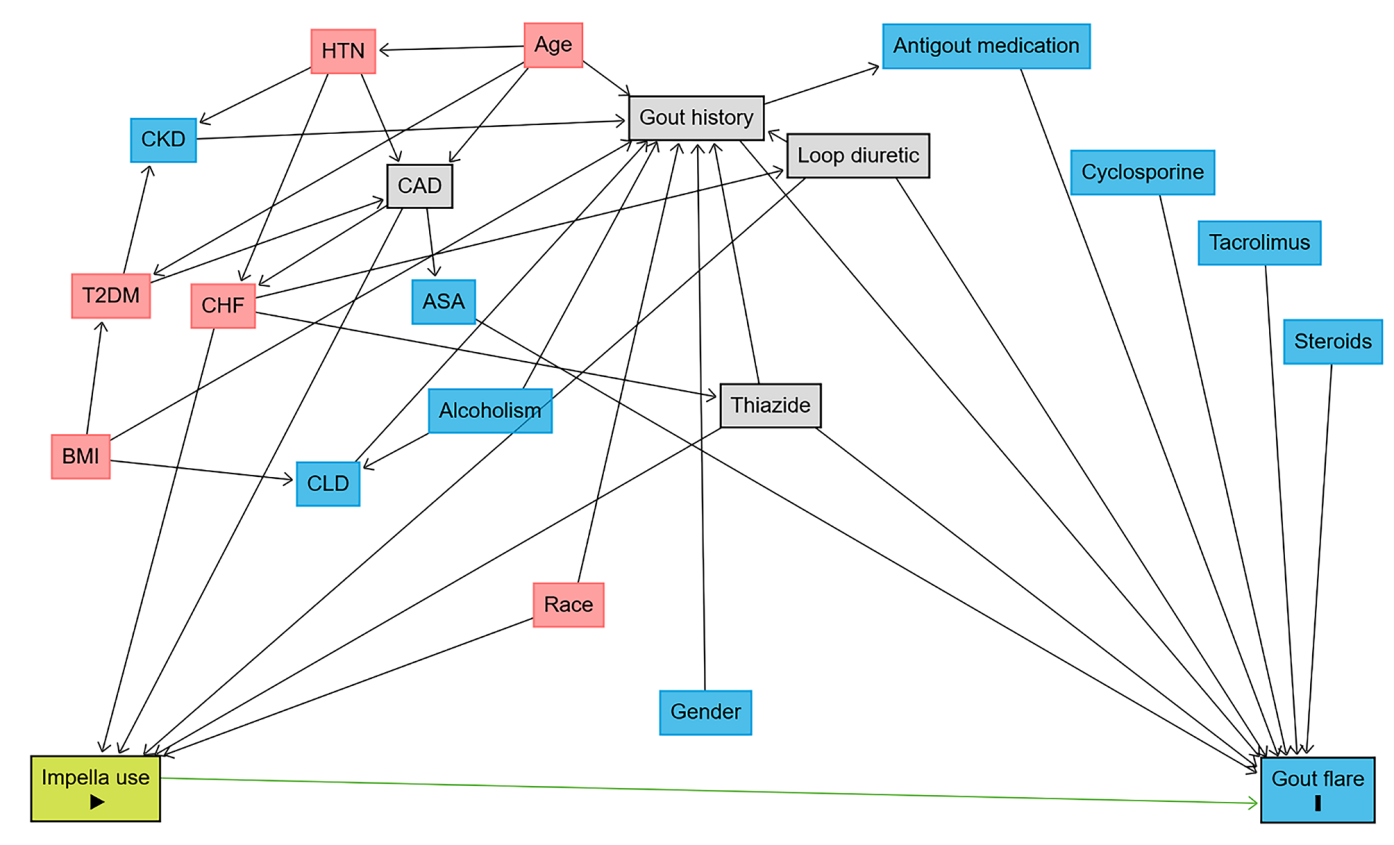
Directed acyclic graph (DAG) demonstrating interaction of various confounders with exposure (Impella use), outcome (Gout flare) and among each other

A directed acyclic graph was built using the ‘dagitty’ R package (DAGitty v3.1 as available on https://dagitty.net) to demonstrate causal inference [8]. Based on the interaction of various confounders with exposure (Impella use), outcome (Gout flare), and among each other, variables appropriate (CAD, gout history, thiazide, and loop diuretic) for minimal sufficient adjustment were identified (Fig. 1). Variables thereby identified were incorporated in propensity score generation using “MatchIt” R package [9]. Propensity scores were calculated for each patient using logistic regression. The matching algorithm employed was the nearest neighbor matching algorithm, and the distance metric utilized was the Generalized Linear Model (GLM). Patients were matched in a 1:1 ratio without replacement and preset caliper distance. Post-matching, an assessment of the covariate balance between the two groups was performed to ensure the success of the propensity score matching. A standardized median difference value below 0.1 was considered to be a negligible imbalance and a value above 0.2 was considered to be a significant imbalance [10]. A covariate balance diagram (Supplementary Fig. 1) was created using the R package “love.plot”. Thereafter a logistic regression model including “weights” set as propensity weight was performed on the matched dataset to demonstrate the causal relationship between impella use and risk of gout flare. Moreover, post-matching residual imbalances were adjusted by a multivariate logistic regression model that included covariates (age, race, gender, CKD, type 2 diabetes, antigout medication use, low dose salicylate, steroids, and tacrolimus) variables with SMD > 0.1 from Table 1.

**Table 1:** Baseline characteristics and outcomes of heart transplant recipients before and after matching categorized by Impella use

	Before Matching			After Matching		
	Non-Impella (*N* = 171)	Impella (*N* = 42)	SMD	Non-Impella (*N* = 42)	Impella (*N* = 42)	SMD
Variables included in minimal sufficient adjustment set (propensity matching and inverse propensity weight)						
Gout	39 (22.8)	17 (40.5)	0.387	16 (38.1)	17 (40.5)	0.049
CAD	72 (42.1)	8 (19)	0.517	8 (19.0)	8 (19.0)	< 0.001
Thiazides	39 (22.8)	19 (45.2)	0.487	17 (40.5)	19 (45.2)	0.096
Loop diuretics	156 (91.2)	33 (78.6)	0.359	33 (78.6)	33 (78.6)	< 0.001
Variables for whom adjustments were not indicated as per DAG						
**Age (years), median (IQR)**	59 (48, 65)	61 (55, 68)	0.371	57 (44, 65)	61 (55, 67)	0.570
**BMI (kg/m** ^**2**^ **); median (IQR)**	28.4 (24.9, 32.3)	28.8 (26, 33.5)	0.006	28 (25, 33)	28.8 (26, 33.5)	0.041
**Race; n (%)**			0.104			0.321
White	111 (64.9)	25 (59.5)		21 (50.0)	25 (59.5)	
African American	49 (28.7)	14 (33.3)		19 (45.2)	14 (33.3)	
Asian Indian	4 (2.3)	1 (2.4)		0 (0)	1 (2.4)	
Caribbean Black	3 (1.8)	1 (2.4)		1 (2.4)	1 (2.4)	
Other	3 (1.8)	0 (0)		1 (2.4)	1 (2.4)	
**Gender; n (%)**			0.551			0.500
Male	118 (69)	38 (90.5)		30 (71.4)	38 (90.5)	
Female	53 (31)	4 (9.5)		12 (28.6)	4 (9.5)	
**Comorbidities; n (%)**						
Hypertension	117 (68.4)	31 (73.8)	0.119	30 (71.4)	31 (73.8)	0.053
CHF	164 (95.9)	39 (92.9)	0.133	39 (92.9)	39 (92.9)	< 0.001
CKD	86 (50.6)	29 (69)	0.390	20 (47.6)	29 (60.0)	0.445
Chronic Liver Disease	15 (8.8)	2 (4.8)	0.160	3 (7.1)	2 (4.8)	0.101
Alcoholism	8 (4.7)	2 (4.8)	0.004	3 (7.1)	2 (4.8)	0.101
DM2	79 (46.2)	24 (57.1)	0.220	16 (38.1)	24 (57.1)	0.389
**Outpatient Antigout Medication; n (%)**	18 (10.5%)	12 (28.6%)	0.467	9 (21.4)	12 (28.6%)	0.166
Colchicine	5 (2.9)	6 (14.3)		4 (9.5)	6 (14.3)	
Allopurinol	15 (8.8)	10 (23.8)		6 (14.3)	10 (23.8)	
Febuxostat	1 (0.6)	2 (4.8)		1 (2.4)	2 (4.8)	
**Inpatient medication; n (%)**						
Low-dose salicylates	97 (56.7)	38 (90.5)	0.829	22 (52.4)	38 (90.5)	0.930
Steroids	136 (79.5)	34 (80.9)	0.036	36 (85.7)	34 (80.9)	0.128
Cyclosporine	9 (5.3)	5 (11.9)	0.239	6 (14.3)	5(11.9)	0.071
Tacrolimus	159 (93)	34 (80.9)	0.363	38 (90.5)	34 (80.9)	0.275
Gout Flare	9 (5.3)	13 (30.9)	0.708	3 (7.1)	13 (30.9)	0.636
**Outcomes**						
Hospital LOS (days); median (IQR)	38.0 (22.0, 65.5)	50.5 (42.0, 76.8)	0.367	38 (26, 55)	50.5 (42.0, 76.8)	0.533
ICU LOS (days); median (IQR)	6.0 (4.0, 13.5)	25.5 (14.3, 42.3)	0.870	6 (4, 11)	25.5 (14.3, 42.3)	0.904
IMV, n(%)	140 (81. 9%)	34 (80.9%)	0.024	32 (76.2)	34 (80.9)	0.116
Mortality, n(%)	26 (15.2%)	4 (9.5%)	0.173	4 (9.5)	4 (9.5)	< 0.001

*Abbreviations: SMD, standardized mean difference; DAG*, *Directed acyclic graph; BMI, body mass index; CAD, coronary artery disease; PUD, peptic ulcer disease; CHF, chronic heart failure; CKD, chronic kidney disease; DM2, diabetes mellitus type 2; LOS, length of stay; ICU, intensive care unit; IMV, invasive mechanical ventilation*

As a supplementary approach, 27 non-Impella patients were matched with 27 impella patients using a 1:1 nearest neighbor propensity-matched cohort with a caliper distance of 0.1. Majority of the variables displayed excellent covariate balance. A logistic regression model including “weights” set as propensity weight was performed on this matched dataset to demonstrate the causal relationship between impella use and risk of gout flare. (Table 2) However, given a low sample size with this approach (*n* = 54), we did not choose to run another multivariate analysis for a few covariates that are otherwise less strong predictors of gout flare.

**Table 2:** Summary table for risk of gout flare as elucidated by different statistical approaches

Summary table for odds of gout flare in Impella supported patients as elucidated by different statistical approaches		
	**Odds ratio**	**95% CI**
Unadjusted Model (*n* = 213)	8.07	3.20–21.26
1:1 nearest neighbor propensity matched without caliper (*n* = 94)	5.83	1.69–27.14
1:1 nearest neighbor propensity matched (without caliper) and MV adjustment of residual confounders (*n* = 94)	4.96	1.19–28.06
1:1 nearest neighbor propensity matched with caliper of 0.1 (*n* = 54) *	4.00	1.03–20.02
Inverse propensity of treatment weighted analysis including all covariates (*n* = 213)	4.21	2.13–8.89

*Abbreviations: CI, Confidence interval; MV, Multivariate*

*Supplementary Tables 1* and* Fig. 1

Finally, to overcome potential limitations set by sample size decrease with 1:1 propensity matching, we also performed inverse propensity of treatment weighting (IPTW) using propensity scores generated using a separate logistic regression that included all demographic, comorbidity, and treatment-related covariates elucidated in Table 1. IPTW was calculated using the formula IPTW = [(Impella use/ Propensity) + {(1– Impella use)/ (1- propensity}]. A separate dataset containing 213 patients with their corresponding IPTW was generated. Thereafter, a separate logistic regression model including “weights” set as ‘inverse propensity of treatment weight’ was performed on the dataset to determine the causal relationship between impella use and risk of gout flare.

In the Impella-supported group, we compared outcome measures based on the diagnosis of gout flares. These outcome measures include in-hospital mortality, hospital LOS, ICU LOS, and IMV and IMV length, and acute kidney injury (AKI). AKI was defined as an increased in creatinine of ≥ 1.5 times baseline or ≥ 0.3 mg/dL rise within 48 h based on KDIGO criteria.

## Results

During the study period, our cohort comprised 213 patients with a median age of 59 years. Among them, 42 (17.8%) received Impella support, while 171 (82.2%) did not. Of the Impella-supported patients, 37 (88%) were assisted with Impella 5.5, and 5 (12%) with Impella CP. Before matching, the Impella-supported and non-Impella groups showed no differences in age, race, and body mass index (BMI), except for a higher percentage of males in the Impella group.

Furthermore, a higher prevalence of gout and chronic kidney disease, coupled with a lower prevalence of coronary artery disease, was observed in the Impella-supported group. They were also more likely to have received antigout medications (colchicine, allopurinol, and febuxostat) prior to hospitalization. Impella-supported patients exhibited a significantly higher utilization of thiazides and low-dose salicylates during hospitalization, while the non-Imeplla group demonstrated a higher utilization of loop diuretics and tacrolimus.

After matching, 42 patients with Impella support were paired with 42 patients without Impella support. Variables identified for minimal sufficient adjustment showed no differences between the matched groups. Demographic characteristics, comorbidities, and medications were compared (Table 1). Significant differences in outcome variables included a higher occurrence of gout flares (30.9% vs. 7.1%; SMD = 0.636), longer ICU LOS (25.5 days vs. 6 days; SMD = 0.904), and hospital LOS (50.5 vs. 38, SMD = 0.533) in the Impella-supported patients.

In the unadjusted analysis, Impella-supported patients had an odds ratio (OR) of 8.07 (95%CI: 3.20-21.26) of having gout flares compared to non-Impella patients. Propensity-matched cohorts, both without a preset caliper and with a preset caliper distance of 0.1 demonstrated OR of 5.83 (95%CI: 1.69–27.14) and 4.00 (95%CI: 1.03–20.02), respectively. Adjusting for residual confounders in the propensity-matched cohort without a preset caliper yielded an OR of 4.96 (95%CI: 1.19–28.06). In an inverse propensity of treatment-weighted analysis, Impella use showed a 4.21-times increased likelihood of having gout flare (OR 4.21, 95%CI: 2.13–8.89)(Table 2).

Comparing outcomes between Impella-supported patients with and without gout flares revealed associations between gout flare and a longer ICU (44 days vs. 19 days, *P* = 0.017) and hospital length of stay (72 vs. 48 days, *P* = 0.032). However, no differences were noted in the prevalence of acute kidney injury, mechanical ventilation days, and in-hospital mortality between the two groups (Table 3).

**Table 3:** Comparison of outcomes associated with gout flare in heart transplant recipients supported with Impella

	Overall (*N* = 42)	No gout flare (*N* = 29)	Gout flare (*N* = 13)	p-value
**Outcomes; n (%)**				
In-hospital mortality	4 (9.5)	4 (14%)	0 (0%)	0.29
Hospital LOS (days); median (IQR)	50 (42, 76)	48 (37, 66)	72 (54, 80)	**0.03**
ICU LOS (days); median (IQR)	25.50 (14, 42)	19 (14, 33)	44 (26, 57)	**0.017**
IMV	34 (81)	23 (80%)	11 (85%)	1.00
IMV length (days)	3 (2, 5)	3 (2, 8)	3 (2, 4)	0.51
Acute Kidney Injury	21 (50)	16 (55%)	5 (38%)	0.51

Abbreviations: LOS, length of stay; IMV, invasive mechanical ventilation

## Discussion

To our knowledge, this study is the first to explore the potential association between Impella support and elevated rates of gout flares during hospitalization. Heart transplanted patients and patients with advanced heart failure frequently have similar risk factors for gout flares, such as chronic kidney disease, history of gout, use of diuretics, low-dose aspirin, and cyclosporine, among others. However, even after matching for traditional risk factors, including gender, comorbidities, and medications, using various propensity-matched approaches, the occurrence of gout flares was significantly higher in patients with Impella support compared to those without. Additionally, it is noteworthy that hospital and ICU LOS was greater in patient with Impella support after matching. This observation could be explained by the necessity for ICU level of care for patients supported with Impella devices.

Although, at this time, there is no clear biological link between Impella support and increased rates of gout flares, there are potential explanations for this observed association. One compelling avenue to explore is the role of hemolysis, a complication frequently encountered in patients with Impella support. While the hemolysis of mature erythrocytes, which lack nucleic acids, does not directly induce hyperuricemia, the erythropoietin response results in increased synthesis of precursors containing nucleic acids that break down during hemolysis, releasing uric acid and increasing uric acid levels [11, 12]. Hemolysis in patients with Impella (a second-generation ventricular assist device) is thought to be caused by shear stress from the friction generated at the bearings of the axial pump. The occurrence of hemolysis in this patient population has been previously reported, with documented rates ranging from 7% to up to 62.5% [13–15]. In a retrospective study including 40 patients managed with Impella support, Badiye et al. [14] found that in 55% of the patients, hemolytic parameters were altered until the time of Impella removal, suggesting persistent hemolysis. Additionally, they observed that 65% of the patients were transfused to maintain an adequate hemoglobin level, with a mean of 7.5 units of red blood cells per patient [14]. However, it is important to note that many of these investigations primarily focused on femoral artery-inserted Impella devices, such as the Impella 2.5 and Impella CP. In contrast, our institution predominantly utilizes axillary Impella devices (Impella 5.5), which are known for their comparatively lower incidence of hemolysis [3, 4, 16]. Nevertheless, it is noteworthy that even Impella 5.5 can occasionally lead to hemolysis, but this observation raises the possibility that factors beyond hemolysis may be contributing to the elevated rates of gout flares in Impella-supported patients.

The consequences of significant bleeding in Impella-supported patients present an additional facet to the association of Impella support and gout flares. Previous studies have reported an incidence of major bleeding ranging from 0.05 to 54% [17]. The risk of bleeding is associated with the requirement for therapeutic anticoagulation and thrombocytopenia [2]. Bleeding can induce volume depletion and decrease the glomerular filtration rate. The resulting lactic acidosis from tissue ischemia may facilitate urate crystallization and can also impair renal uric acid excretion as lactate competes with urate in the proximal tubule [18]. Additionally, volume depletion can increase net uric acid reabsorption by the proximal tubule, which can further induce gout flares [19]. Moreover, a common indication for Impella support is cardiogenic shock. This state of shock can also lead to lactic acidosis, volume depletion, and renal impairment, potentially playing a role in the development of gout flares. Furthermore, the anemia resulting from both hemolysis and bleeding could impair oxidative metabolism, which may lead to the development of hyperuricemia and gout flare [20, 21]. While these explanations provide valuable insight into the potential mechanisms at play, further investigation is essential to confirm this.

The occurrence of gout flares can significantly impact the mobilization and overall comfort of hospitalized patients undergoing heart transplantation. Both pre-and post-transplant mobilization and participation in rehabilitation programs have demonstrated significant short and long-term benefits for heart transplant recipients [22]. Additionally, our study shows a potential association between gout flares and prolonged hospital and ICU stays in Impella-supported patients undergoing heart transplantation. The median hospital and ICU LOS of Impella-supported patients with gout flares was 24 days and 25 days longer, respectively, compared to those without gout flares. These findings align with previous research that has consistently reported similar trends in various medical contexts [23–25]. These observations could be attributed to the need for additional diagnostic studies, inpatient consultations, and therapies, which may result in extended hospital stays. In addition to patient comfort and well-being, increased LOS and use of diagnostic testing contribute to increased healthcare costs [26]. However, it is crucial to recognize that the presence of a gout flare does not inherently imply a causal association with an increased hospital and ICU LOS. While gout flares have independently shown an association with prolonged hospital stays [20], it’s noteworthy that an extended LOS also elevates the risk of gout flares. Furthermore, unexplored factors, including post-operative complications not accounted for in our study, may contribute to an extended length of stay. The intricate and multifactorial nature of the association between the development of gout flares and prolonged hospital and ICU stays in this patient population underscores the need for further investigations. Additional studies are essential to comprehensively evaluate the potential morbidity, impact on short and long-term outcome, and the associated costs linked to the occurrence of gout flares.

Our study is strengthened by the multiple statistical approaches adopted while evaluating the research question, in an attempt to control the effect of potential confounders. However, several limitations should be considered when interpreting the findings of our study. Firstly, our study design is retrospective in nature, which inherently introduces the potential for selection bias and limits our ability to establish true causal relationships. Furthermore, determining the occurrence of gout flares based on their documentation in clinical notes could lead to potential underreporting or misclassification, given the variability in documentation practices. Secondly, being a single-center study with a relatively small sample size might limit the generalizability of our results to broader populations. The small sample size also increased the susceptibility to residual confounding due to limited available matches. Additionally, we encountered limitations in the availability of comprehensive data, particularly regarding uric acid levels, presence of subcutaneous tophi, duration and quality of pre-hospital urate-lowering therapy, CKD stage, gout flare prophylaxis, hemodynamic parameters, the documentation of post-operative complications, and other variables that could influence the development of gout flares and outcomes. These limitations collectively underscore the need for larger, prospective, and multi-center studies to validate and extend our findings while also considering a wider array of variables to provide a more comprehensive understanding of the association between Impella support and gout flares.

## Conclusion

Our study is the first to identify the potential association between Impella support, particularly the Impella 5.5 model, and increased rates of gout flares in hospitalized patients. Considering the potential morbidity and financial implications associated with the development of gout flares, future studies are required to confirm this association and further elucidate the biological pathways of Impellasupport and gout flares. It is imperative to consider introducing appropriate measures to prevent and promptly manage gout flares in Impella-supported patients.

### Electronic supplementary material

Below is the link to the electronic supplementary material.

Supplementary Material 1

## Data Availability

Access to the data used in this research is available upon request to the corresponding authors. Patients’ privacy concern, HIPPA regulation, and institutional rules will be strictly considered while providing data.

## Abbreviations

BMI: Body mass index
ICU: Intensive care unit
IMV: Invasive mechanical ventilation
LOS: Length of Stay
