# Supplementary material for: Association between Impella device support and elevated rates of gout flares: a retrospective propensity-matched study
Source: BMC Rheumatol. 2024 Feb 29;8:9. doi: 10.1186/s41927-024-00380-z (PMC10902952; doi:10.1186/s41927-024-00380-z)
Supplement: Supplementary file 1 — Supplementary Material 1 [file 41927_2024_380_MOESM1_ESM.docx]

**Supplementary Table 1: Baseline characteristics and outcomes of heart transplant recipients before and after matching categorized by Impella use in a 1:1 nearest neighbor propensity-matched cohort with a caliper distance of 0.1.**

|  | Before Matching (n=213) | | | After Matching (n=54) | | |
| --- | --- | --- | --- | --- | --- | --- |
|  | Non-Impella  (N = 171) | Impella  (N = 42) | SMD | Non-Impella  (N = 27) | Impella  (N = 27) | SMD |
| **Age (years), median (IQR)** | 59 (48, 65) | 61 (55, 68) | 0.371 | 60 (53, 65) | 62 (55, 68) | 0.243 |
| **BMI (kg/m^2^); median (IQR)** | 28.4 (24.9, 32.3) | 28.8 (26, 33.5) | 0.006 | 28 (26, 33) | 29 (26, 34) | 0.101 |
| **Race; n (%)** |  |  | 0.104 |  |  | 0.298 |
| White | 111 (64.9) | 25 (59.5) |  | 15 (55.6) | 17 (63.0) |  |
| African American | 49 (28.7) | 14 (33.3) |  | 11 (40.7) | 10 (37.0) |  |
| Asian Indian | 4 (2.3) | 1 (2.4) |  | 0 (0.0) | 0 (0.0) |  |
| Caribbean Black | 3 (1.8) | 1 (2.4) |  | 0 (0.0) | 0 (0.0) |  |
| Other | 3 (1.8) | 0 (0) |  | 1 (3.7) | 0 (0.0) |  |
| **Gender; n (%)** |  |  | 0.551 |  |  | <0.001 |
| Male | 118 (69) | 38 (90.5) |  | 23 (85.2) | 23 (85.2) |  |
| Female | 53 (31) | 4 (9.5) |  | 4 (14.8) | 4 (14.8) |  |
| **Comorbidities; n (%)** |  |  |  |  |  |  |
| Hypertension | 117 (68.4) | 31 (73.8) | 0.119 | 24 (88.9) | 21 (77.8) | 0.302 |
| CAD | 72 (42.1) | 8 (19) | 0.517 | 7 (25.9) | 8 (29.6) | 0.083 |
| CHF | 164 (95.9) | 39 (92.9) | 0.133 | 24 (88.9) | 25 (92.6) | 0.128 |
| CKD | 86 (50.6) | 29 (69) | 0.390 | 19 (70.4) | 18 (66.7) | 0.080 |
| Gout | 39 (22.8) | 17 (40.5) | 0.387 | 13 (48.1) | 12 (44.4) | 0.074 |
| Chronic Liver Disease | 15 (8.8) | 2 (4.8) | 0.160 | 2 (7.4) | 2 (7.4) | <0.001 |
| Alcoholism | 8 (4.7) | 2 (4.8) | 0.004 | 1 (3.7) | 1 (3.7) | <0.001 |
| DM2 | 79 (46.2) | 24 (57.1) | 0.220 | 16 (59.3) | 16 (59.3) | <0.001 |
| **Outpatient Antigout Medication; n (%)** | 18 (10.5%) | 12 (28.6%) | 0.467 | 7 (25.9) | 8 (29.6 | 0.083 |
| Colchicine | 5 (2.9) | 6 (14.3) |  | 1 (3.7) | 4 (14.8) |  |
| Allopurinol | 15 (8.8) | 10 (23.8) |  | 6 (22.2) | 7 (25.9) |  |
| Febuxostat | 1 (0.6) | 2 (4.8) |  | 1 (3.7) | 1 (3.7) |  |
| **Inpatient medication; n (%)** |  |  |  |  |  |  |
| Thiazides | 39 (22.8) | 19 (45.2) | 0.487 | 9 (33.3) | 9 (33.3) | <0.001 |
| Loop diuretics | 156 (91.2) | 33 (78.6) | 0.359 | 23 (85.2) | 23 (85.2) | <0.001 |
| Low-dose salicylates | 97 (56.7) | 38 (90.5) | 0.829 | 23 (85.2) | 23 (85.2) | <0.001 |
| Steroids | 136 (79.5) | 34 (80.9) | 0.036 | 20 (74.1) | 21 (77.8) | 0.087 |
| Cyclosporine | 9 (5.3) | 5 (11.9) | 0.239 | 4 (14.8) | 3 (11.1) | 0.110 |
| Tacrolimus | 159 (93) | 34 (80.9) | 0.363 | 22 (81.5) | 22 (81.5) | <0.001 |
| Gout Flare | 9 (5.3) | 13 (30.9) | 0.708 | 3 (11.1) | 9 (33.3) | 0.555 |
| **Outcomes** |  |  |  |  |  |  |
| Hospital LOS (days); median (IQR) | 38.0 (22.0, 65.5) | 50.5 (42.0, 76.8) | 0.367 | 52.0 (35.0, 65.0) | 49.0 (38.0, 70.0) | 0.094 |
| ICU LOS (days); median (IQR) | 6.0 (4.0, 13.5) | 25.5 (14.3, 42.3) | 0.870 | 9.0 (4.0, 20.0) | 22.0 (14.0, 36.0) | 0.669 |
| IMV, n(%) | 140 (81. 9%) | 34 (80.9%) | 0.024 | 23 (85.2) | 24 (88.9) | 0.110 |
| Mortality, n(%) | 26 (15.2%) | 4 (9.5%) | 0.173 | 0 (0.0) | 3 (11.1) | 0.500 |

*Abbreviations: SMD, standardized mean difference; DAG,* *Directed acyclic graph; BMI, body mass index; CAD, coronary artery disease; PUD, peptic ulcer disease; CHF, chronic heart failure; CKD, chronic kidney disease; DM2, diabetes mellitus type 2; LOS, length of stay; ICU, intensive care unit; IMV, invasive mechanical ventilation*

**Supplementary Figure 1: Love plot demonstrating covariate balance after 1:1 nearest neighbor propensity matching with caliper distance of 0.1. All covariates related to demographic, comorbidity, and treatment, as elucidated in Table 1, were included.**

**
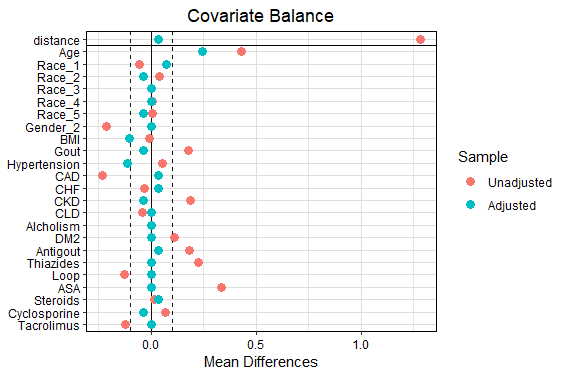
**

*Abbreviations: BMI, body mass index; CAD, coronary artery disease; CHF, chronic heart failure; CKD, chronic kidney disease; DM2, diabetes mellitus type 2; CLD, Chronic liver disease; Gout, History of Gout; ASA, Acetyl salicylic acid (low dose)*
